# Trends and Distribution of In-Hospital Mortality Among Pregnant and Postpartum Individuals by Pregnancy Period

**DOI:** 10.1001/jamanetworkopen.2022.24614

**Published:** 2022-07-29

**Authors:** Lindsay K. Admon, Nicole D. Ford, Jean Y. Ko, Cynthia Ferre, Charlan D. Kroelinger, Katy B. Kozhimannil, Elena V. Kuklina

**Affiliations:** 1 Department of Obstetrics and Gynecology and Institute for Healthcare Policy and Innovation, University of Michigan, Ann Arbor; 2 Division of Reproductive Health, National Center for Chronic Disease Prevention and Health Promotion, Centers for Disease Control and Prevention, Atlanta, Georgia; 3 Department of Health Policy and Management, University of Minnesota School of Public Health, Minneapolis

## Abstract

This cross-sectional study investigates trends in death rates and proportion of deaths by pregnancy period among pregnant and postpartum individuals from 1994 to 2019.

## Introduction

Nearly half of pregnancy-related deaths happen during inpatient hospitalizations, more than one-quarter of which may occur after childbirth, and the pregnancy-related mortality ratio is increasing in the hospital setting.^1,2^ This study aimed to examine long-term trends in inpatient death rates among pregnant and postpartum individuals and proportion of deaths by pregnancy period (antenatal, delivery, and postpartum).

## Methods

The Centers for Disease Control and Prevention determined that this cross-sectional study was exempt from institutional review board review and informed consent because this was a secondary data analysis of deidentified data. This study followed the STROBE reporting guideline. Using data from the National Inpatient Sample for 1994 to 2015 and 2017 to 2019,^3^ we examined patterns of inpatient mortality during pregnancy-associated hospitalizations. We hierarchically categorized hospitalizations by pregnancy period (delivery, postpartum, and antenatal) using *International Classification of Diseases, Ninth Revision, Clinical Modification *(*ICD-9-CM*) and* International Statistical Classification of Diseases, Tenth Revision, Clinical Modification* (*ICD-10-CM*) and diagnosis-related group codes (eTables 1-2 in the Supplement). Inpatient mortality was identified based on discharge disposition, which was missing in less than 0.01% of observations. For 1994 to 2015, we assessed temporal trends in the rate of inpatient mortality by pregnancy period and their statistical significance using orthogonal polynomial coefficients calculated recursively for linear trend testing. Given the *ICD-CM* coding transition in the third quarter of 2015, we excluded 2016 data, and 1994 to 2015 and 2017 to 2019 periods were examined separately. Using combined 2017 to 2019 data, we described the proportion of hospitalizations and inpatient deaths by pregnancy period.

## Results

**Figure 1.**
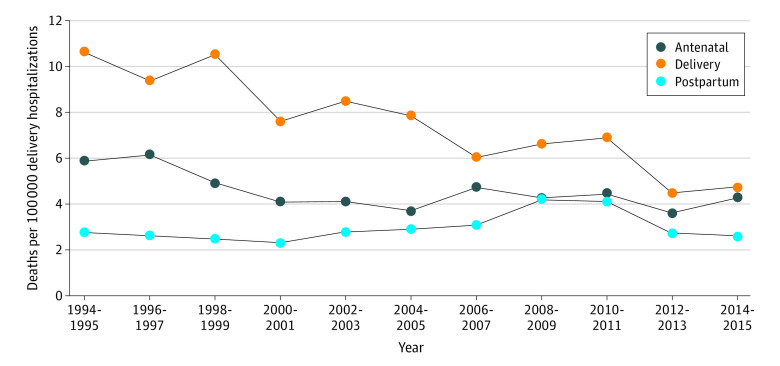
Trends in In-Hospital Mortality Among Pregnancy Hospitalizations Data are from the National Inpatient Sample. Temporal trends in the rate of inpatient mortality associated with each pregnancy period were assessed using orthogonal polynomial coefficients calculated recursively. *P* value was calculated for these linear trend tests. All analyses were performed using SAS statistical software version 9.4 (SAS Institute), with 2-sided tests and an α = .05 threshold.

**Figure 2.**
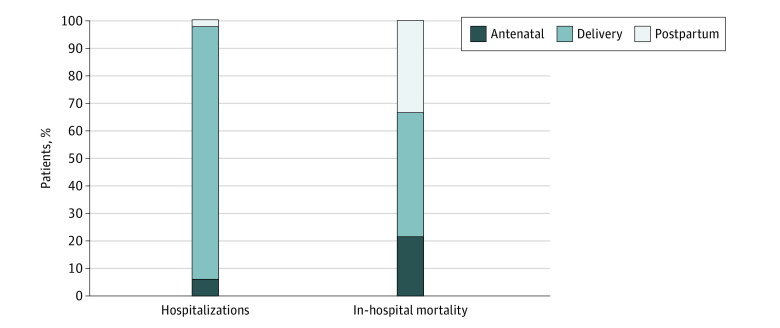
Distribution of Hospitalizations and In-Hospital Deaths by Pregnancy Period Data are from 2017 to 2019.

Between 1994 and 2015, an estimated 12 654 inpatient deaths occurred among pregnant and postpartum individuals with a mean (standard error [SE]) age of 29.37 (0.14) years among 84 181 338 hospitalizations (mean [SE] age, 27.47 [0.001] years). Regression analyses revealed that inpatient deaths during delivery hospitalizations decreased from 10.6 (95% CI, 8.3 to 12.9) deaths per 100 000 delivery hospitalizations (788 inpatient deaths among 7 423 264 delivery hospitalizations) to 4.7 (95% CI, 3.5 to 5.8) deaths per 100 000 delivery hospitalizations (310 inpatient deaths among 6 661 065 delivery hospitalizations) between 1994 to 1995 and 2014 to 2015 (absolute change = –5.9 [95% CI, –8.5 to –3.4] deaths per 100 000 delivery hospitalizations; *P* < .001) (Figure 1). The rate of inpatient deaths in antenatal and postpartum periods remained unchanged between 1994 to 1995 and 2014 to 2015 (Figure 1). From 1994 to 2015, overall rates of inpatient mortality for antenatal and postpartum hospitalizations were 4.5 (95% CI, 4.1 to 4.9) and 3.0 (95% CI, 2.7 to 3.3) deaths per 100 000 hospital deliveries, respectively. An estimated 1480 inpatient deaths (mean [SE] age, 31.31 [0.44] years) and 10 898 224 delivery hospitalizations (mean [SE] age, 28.89 [<0.001] years) were identified in 2017 to 2019; rates of death for antenatal, delivery, and postpartum hospitalizations were, respectively, 2.9 (95% CI, 2.2 to 3.7), 6.1 (95% CI, 5.1 to 7.2 ), and 4.5 (95% CI, 3.6 to 5.4) deaths per 100 000 hospital deliveries. In 2017 to 2019, antenatal and postpartum hospitalizations accounted for 6.2% and 2.3% of perinatal hospitalizations, yet 21.6% and 33.1% of inpatient deaths occurred during these periods, respectively (Figure 2).

## Discussion

This cross-sectional study found that between 1994 to 1995 and 2014 to 2015, the rate of inpatient death during delivery hospitalizations decreased by 6 percentage points, while rates of inpatient death during antenatal and postpartum hospitalizations did not change. During 2017 to 2019, antenatal and postpartum hospitalizations accounted for less than 10% of inpatient stays but more than half of inpatient deaths of pregnant and postpartum individuals.

Resources directed toward improving quality of care at obstetric delivery have been associated with decreased rates of severe morbidity^4^ and may be associated with decreased mortality identified during delivery hospitalizations. Clinical and policy efforts, however, may need to be additionally directed toward antenatal and postpartum hospitalizations and structural factors associated with increased risk for adverse outcomes before and after delivery.^5^

This study’s limitations include modeling assumptions and reliance on cross-sectional, administrative data, which may be subject to coding inaccuracies and misclassification. Future research is needed to identify and address inequities in inpatient maternal morbidity and mortality stratified by individual, community, and structural factors.

## Notes

Supplement. **eTable 1.** *International Classification of Diseases, Ninth Revision, Clinical Modification* (*ICD-9-CM*) Codes Used to Identify Pregnancy-Associated Hospitalizations and Pregnancy Period **eTable 2.** *International Statistical Classification of Diseases, Tenth Revision, Clinical Modification* (*ICD-10-CM*) Codes to Identify Pregnancy-Associated Hospitalizations and Pregnancy Period
