# Data Error in Discussion

**DOI:** 10.1001/jamanetworkopen.2022.32512

**Published:** 2022-08-26

In the Research Letter titled “Trends and Distribution of In-Hospital Mortality Among Pregnant and Postpartum Individuals by Pregnancy Period,”^1^ published July 29, 2022, there was an error in a number in the Discussion. The decrease in the rate of inpatient death during delivery hospitalizations between 1994 to 1995 and 2014 to 2015 should have been described as 56%. This article has been corrected.^1^
