# Supplementary material for: Trends and Distribution of In-Hospital Mortality Among Pregnant and Postpartum Individuals by Pregnancy Period
Source: JAMA Netw Open. 2022 Jul 29;5(7):e2224614. doi: 10.1001/jamanetworkopen.2022.24614 (PMC9338405; doi:10.1001/jamanetworkopen.2022.24614)
Supplement: Supplement. — eTable 1. International Classification of Diseases, Ninth Revision, Clinical Modification (ICD-9-CM) Codes Used to Identify Pregnancy-Associated Hospitalizations and Pregnancy Period eTable 2. International Statistical Classification of Diseases, Tenth Revision, Clinical Modification (ICD-10-CM) Codes to Identify Pregnancy-Associated Hospitalizations and Pregnancy Period [file jamanetwopen-e2224614-s001.pdf]

Supplemental Online Content

Admon LK, Ford ND, Ko JY, et al. Trends and distribution of in-hospital mortality among pregnant and postpartum individuals by pregnancy period. *JAMA Netw Open*. 2022;5(7):e2224614. doi:10.1001/jamanetworkopen.2022.24614

eTable 1. *International Classification of Diseases, Ninth Revision, Clinical Modification* (*ICD-9-CM*) Codes Used to Identify Pregnancy-Associated Hospitalizations and Pregnancy Period
eTable 2. *International Statistical Classification of Diseases, Tenth Revision, Clinical Modification* (*ICD-10-CM*) Codes to Identify Pregnancy-Associated Hospitalizations and Pregnancy Period

This supplemental material has been provided by the authors to give readers additional information about their work.

© 2022 Admon LK et al. *JAMA Network Open.*

eTable 1. *International Classification of Diseases, Ninth Revision, Clinical Modification* (*ICD-9-CM*) Codes Used to Identify Pregnancy-Associated Hospitalizations and Pregnancy Period

	**ICD-9**
**Pregnancy-associated hospitalizations**	
Diagnosis codes	630x-677x, V22, V23, V24, V27, V28, 7923
Procedure codes	72x-75x
DRG	370, 371, 372, 373, 374, 375, 378, 379, 380, 381, 382, 383, 384, 376, 377 (for years 1997-2007)<br><br>765, 766, 767, 768, 774, 775, 770, 777, 778, 779, 780, 781, 782, 769, 776 (for years 2008-2015)
**Delivery inclusion**	
Diagnosis codes	V27, 650
Procedure codes	720, 721, 7221, 7229, 7231, 7239, 724, 7251, 7252, 7253, 7254, 726, 7271, 7279, 728, 729, 7322, 7359, 736, 740, 741, 742, 744, 7499
DRG	370, 371, 372, 373, 374, 375 (for years 1997-2007)<br>765, 766, 767, 768, 774, 775 (for years 2008-2015)
**Delivery exclusion**	
Diagnosis codes	630, 631, 632, 633, 634, 635, 636, 637, 638, 639
Procedure codes	6901, 6951, 7491, 750
**Postpartum**	
Diagnosis codes	V24<br>Pregnancy-associated diagnosis codes with fifth digit of ‘4’
DRG	376, 377 (for years 1997-2007)<br>769, 776 (for years 2008-2015)
**Antenatal**	
Diagnosis codes	V22, V23, V28<br>Diagnosis codes with fifth digit of ‘3’ among pregnancy-associated hospitalizations
DRG	378, 379, 380, 381, 382, 383, 384 (for years 1997-2007)<br>770, 777, 778, 779, 780, 781, 782 (for years 2008-September 2015)

References:

Kuklina E V., Whiteman MK, Hillis SD, et al. An enhanced method for identifying obstetric deliveries: Implications for estimating maternal morbidity. *Matern Child Health J*. 2008;12(4):469-477. doi:10.1007/S10995-007-0256-6/TABLES/5.

Kuklina E V., Tong X, Bansil P, George MG, Callaghan WM. Trends in pregnancy hospitalizations that included a stroke in the United States from 1994 to 2007: Reasons for concern? *Stroke*. 2011;42(9):2564-2570. doi:10.1161/STROKEAHA.110.610592

© 2022 Admon LK et al. *JAMA Network Open.*

eTable 2. *International Statistical Classification of Diseases, Tenth Revision, Clinical Modification* (*ICD-10-CM*) Codes to Identify Pregnancy-Associated Hospitalizations and Pregnancy Period

	**ICD-10**
**Pregnancy-associated hospitalizations**	
Diagnosis codes	
Delivery	Z370, Z371, Z372, Z373, Z374, Z3750, Z3751, Z3752, Z3753, Z3754, Z3759, Z3760, Z3761, Z3762, Z3763, Z3764, Z3769, Z377, Z379, O80, O82, O7582
Postpartum encounters	Z390, Z391, Z392
Maternal complications in the puerperium	O1003 ,O1013 ,O1023 ,O1033 ,O1043 ,O1093 ,O115 ,O1205 ,O1215 ,O1225 ,O135 ,O1405 ,O1415 ,O1425 ,O1495 ,O152 ,O165 ,O2403 ,O2413 ,O2433 ,O24430 ,O24434 ,O24435 ,O24439 ,O2483 ,O2493 ,O253 ,O2663 ,O2673 ,O870 ,O871 ,O872 ,O873 ,O874 ,O878 ,O879 ,O8803 ,O8813 ,O8823 ,O8833 ,O8883 ,O8901 ,O8909 ,O891 ,O892 ,O893 ,O894 ,O895 ,O896 ,O898 ,O899 ,O9081 ,O9089 ,O909 ,O9102 ,O9112 ,O9122 ,O9803 ,O9813 ,O9823 ,O9833 ,O9843 ,O9853 ,O9863 ,O9873 ,O9883 ,O9893 ,O9903 ,O9913 ,O99215 ,O99285 ,O99315 ,O99325 ,O99335 ,O99345 ,O99355 ,O9943 ,O9953 ,O9963 ,O9973 ,O99815 ,O99825 ,O99835 ,O99845 ,O9989 ,O9A13 ,O9A23 ,O9A33 ,O9A43 ,O9A53 ,O1033 ,O1043 ,O1093 ,O115 , O903, O904, O85, O860, O8611, O8612, O8613, O8619, O8620, O8621, O8622, O8629, O864, O8681,O8689
Antenatal encounter	O0900 ,O0901 ,O0902 ,O0903 ,O0910 ,O0911 ,O0912 ,O0913 ,O09211 ,O09212 ,O09213 ,O09219 ,O09291 ,O09291 ,O09291 ,O09292 ,O09293 ,O09299 ,O0930 ,O0931 ,O0932 ,O0933 ,O0940 ,O0941 ,O0942 ,O0943 ,O09511 ,O09512 ,O09513 ,O09519 ,O09521 ,O09522 ,O09523 ,O09529 ,O09611 ,O09612 ,O09613 ,O09619 ,O09621 ,O09622 ,O09623 ,O09629 ,O0970 ,O0971 ,O0972 ,O0973 ,O09811 ,O09812 ,O09813 ,O09819 ,O09821 ,O09822 ,O09823 ,O09829 ,O09891 ,O09892 ,O09893 ,O09899 ,O0990 ,O0991 ,O0992 ,O0993 ,O09A0 ,O09A1 ,O09A2 ,O09A3 ,Z331 ,Z3400 ,Z3401 ,Z3402 ,Z3403 ,Z3480 ,Z3481 ,Z3482 ,Z3483 ,Z3490 ,Z3491 ,Z3492 ,Z3493 ,Z36 ,Z3201 ,Z332 , Z333
Antenatal complications	O10011 ,O10012 ,O10013 ,O10019 ,O10111 ,O10112 ,O10113 ,O10119 ,O10211 ,O10212 ,O10213 ,O10219 ,O10311 ,O10312 ,O10313 ,O10319 ,O10411 ,O10412 ,O10413 ,O10419 ,O10911 ,O10912 ,O10913 ,O10919 ,O111 ,O112 ,O113 ,O119 ,O1200 ,O1201 ,O1202 ,O1203 ,O1210 ,O1211 ,O1212 ,O1213 ,O1220 ,O1221 ,O1222 ,O1223 ,O131 ,O132 ,O133 ,O139 ,O1400 ,O1402 ,O1403 ,O1410 ,O1412 ,O1413 ,O1420 ,O1422 ,O1423 ,O1490 ,O1492 ,O1493 ,O1500 ,O1502 ,O1503 ,O151 ,O159 ,O161 ,O162 ,O163 ,O169 ,O200 ,O208 ,O209 ,O210 ,O211 ,O212 ,O218 ,O219 ,O2200 ,O2201 ,O2202 ,O2203 ,O2210 ,O2211 ,O2212 ,O2213 ,O2220 ,O2221 ,O2222 ,O2223 ,O2230 ,O2231 ,O2232 ,O2233 ,O2240 ,O2241 ,O2242 ,O2243 ,O2250 ,O2251 ,O2252 ,O2253 ,O228X1 ,O228X2 ,O228X3 ,O228X9 ,O2290 ,O2291 ,O2292 ,O2293 ,O2300 ,O2301 ,O2302 ,O2303 ,O2310 ,O2311 ,O2312 ,O2313 ,O2320 ,O2321 ,O2322 ,O2323 ,O2330 ,O2331 ,O2332 ,O2333 ,O2340 ,O2341 ,O2342 ,O2343 ,O23511 ,O23512 ,O23513 ,O23519 ,O23521 ,O23522 ,O23523 ,O23529 ,O23591 ,O23592 ,O23593 ,O23599 ,O2390 ,O2391 ,O2392 ,O2393 ,O24011 ,O24012 ,O24013 ,O24019 ,O24111 ,O24112 ,O24113 ,O24119 ,O24311 ,O24312 ,O24313 ,O24319 ,O24410 ,O24414 ,O24415 ,O24419 ,O24811 ,O24812 ,O24813 ,O24819 ,O24911 ,O24912 ,O24913 ,O24919 ,O2510 ,O2511 ,O2512 ,O2513 ,O2600 ,O2601 ,O2602 ,O2603 ,O2610 ,O2611 ,O2612 ,O2613 ,O2620 ,O2621 ,O2622 ,O2623 ,O2630 ,O2631 ,O2632 ,O2633 ,O2640 ,O2641 ,O2642 ,O2643 ,O2650 ,O2651 ,O2652 ,O2653 ,O26611 ,O26612 ,O26613 ,O26619 ,O26711 ,O26712 ,O26713 ,O26719 O10011 ,O10012 ,O10013 ,O10019 ,O10111 ,O10112 ,O10113 ,O10119 ,O10211 ,O10212 ,O10213 ,O10219 ,O10311 ,O10312 ,O10313

© 2022 Admon LK et al. *JAMA Network Open.*

**Pregnancy-associated hospitalizations**	**ICD-10**
Antenatal complications continued	,O10319 ,O10411 ,O10412 ,O10413 ,O10419 ,O10911 ,O10912 ,O10913 ,O10919 ,O111 ,O112 ,O113 ,O119 ,O1200<br>,O1201 ,O1202 ,O1203 ,O1210 ,O1211 ,O1212 ,O1213 ,O1220 ,O1221 ,O1222 ,O1223 ,O131 ,O132,O133 ,O139<br>,O1400 ,O1402 ,O1403 ,O1410 ,O1412 ,O1413 ,O1420 ,O1422 ,O1423 ,O1490 ,O1492 ,O1493 ,O1500 ,O1502 ,O1503<br>,O151 ,O159 ,O161 ,O162 ,O163 ,O169 ,O200 ,O208 ,O209 ,O210 ,O211 ,O212 ,O218 ,O219 ,O2200 ,O2201 ,O2202<br>,O2203 ,O2210 ,O2211 ,O2212 ,O2213 ,O2220 ,O2221 ,O2222 ,O2223 ,O2230 ,O2231 ,O2232 ,O2233 ,O2240 ,O2241<br>,O2242 ,O2243 ,O2250 ,O2251 ,O2252 ,O2253 ,O228X1 ,O228X2 ,O228X3 ,O228X9 ,O2290 ,O2291 ,O2292 ,O2293<br>,O2300 ,O2301 ,O2302 ,O2303 ,O2310 ,O2311 ,O2312 ,O2313 ,O2320 ,O2321 ,O2322 ,O2323 ,O2330 ,O2331 ,O2332<br>,O2333 ,O2340 ,O2341 ,O2342 ,O2343 ,O23511 ,O23512 ,O23513 ,O23519 ,O23521 ,O23522 ,O23523 ,O23529<br>,O23591 ,O23592 ,O23593 ,O23599 ,O2390 ,O2391 ,O2392 ,O2393 ,O24011 ,O24012 ,O24013 ,O24019 ,O24111<br>,O24112 ,O24113 ,O24119 ,O24311 ,O24312 ,O24313 ,O24319 ,O24410 ,O24414 ,O24415 ,O24419 ,O24811 ,O24812<br>,O24813 ,O24819 ,O24911 ,O24912 ,O24913 ,O24919 ,O2510 ,O2511 ,O2512 ,O2513 ,O2600 ,O2601 ,O2602 ,O2603<br>,O2610 ,O2611 ,O2612 ,O2613 ,O2620 ,O2621 ,O2622 ,O2623 ,O2630 ,O2631 ,O2632 ,O2633 ,O2640 ,O2641 ,O2642<br>,O2643 ,O2650 ,O2651 ,O2652 ,O2653 ,O26611 ,O26612 ,O26613 ,O26619 ,O26711 ,O26712 ,O26713 ,O26719<br>,O26811 ,O26812 ,O26813 ,O26819 ,O26821 ,O26822 ,O26823 ,O26829 ,O26831 ,O26832 ,O26833 ,O26839 ,O26841<br>,O26842 ,O26843 ,O26849 ,O26851 ,O26852 ,O26853 ,O26859 ,O2686 ,O26872 ,O26873 ,O26879 ,O26891 ,O26892<br>,O26893 ,O26899 ,O2690 ,O2691 ,O2692 ,O2693 ,O280 ,O281 ,O282 ,O283 ,O284 ,O285 ,O288 ,O289 ,O29011<br>,O29012 ,O29013 ,O29019 ,O29021 ,O29022 ,O29023 ,O29029 ,O29091 ,O29092 ,O29093 ,O29099 ,O29111 ,O29112<br>,O29113 ,O29119 ,O29121 ,O29122 ,O29123 ,O29129 ,O29191 ,O29192 ,O29193 ,O29199 ,O29211 ,O29212 ,O29213<br>,O29219 ,O29291 ,O29292 ,O29293 ,O29299 ,O293X1 ,O293X2 ,O293X3 ,O293X9 ,O2940 ,O2941 ,O2942 ,O2943<br>,O295X1 ,O295X2 ,O295X3 ,O295X9 ,O2960 ,O2961 ,O2962 ,O2963 ,O298X1 ,O298X2 ,O298X3 ,O298X9 ,O2990<br>,O2991 ,O2992 ,O2993 ,O30001 ,O30002 ,O30003 ,O30009 ,O30011 ,O30012 ,O30013 ,O30019 ,O30021 ,O30022<br>,O30023 ,O30029 ,O30031 ,O30032 ,O30033 ,O30039 ,O30041 ,O30042 ,O30043 ,O30049 ,O30091 ,O30092 ,O30093<br>,O30099 ,O30101 ,O30102 ,O30103 ,O30109 ,O30111 ,O30112 ,O30113 ,O30119 ,O30121 ,O30122 ,O30123 ,O30129<br>,O30191 ,O30192 ,O30193 ,O30199 ,O30201 ,O30202 ,O30203 ,O30209 ,O30211 ,O30212 ,O30213 ,O30219 ,O30221<br>,O30222 ,O30223 ,O30229 ,O30291 ,O30292 ,O30293 ,O30299 ,O30801 ,O30802 ,O30803 ,O30809 ,O30811 ,O30812<br>,O30813 ,O30819 ,O30821 ,O30822 ,O30823 ,O30829 ,O30891 ,O30892 ,O30893 ,O30899 ,O3090 ,O3091 ,O3092<br>,O3093 ,O3100X0 ,O3100X1 ,O3100X2 ,O3100X3 ,O3100X4 ,O3100X5 ,O3100X9 ,O3101X0 ,O3101X1 ,O3101X2<br>,O3101X3 ,O3101X4 ,O3101X5 ,O3101X9 ,O3102X0 ,O3102X1 ,O3102X2 ,O3102X3 ,O3102X4 ,O3102X5 ,O3102X9<br>,O3103X0 ,O3103X1 ,O3103X2 ,O3103X3 ,O3103X4 ,O3103X5 ,O3103X9 ,O3110X0 ,O3110X1 ,O3110X2 ,O3110X3<br>,O3110X4 ,O3110X5 ,O3110X9 ,O3111X0 ,O3111X1 ,O3111X2 ,O3111X3 ,O3111X4 ,O3111X5 ,O3111X9 ,O3112X0<br>,O3112X1 ,O3112X2 ,O3112X3 ,O3112X4 ,O3112X5 ,O3112X9 ,O3113X0 ,O3113X1 ,O3113X2 ,O3113X3 ,O3113X4<br>,O3113X5 ,O3113X9 ,O3120X0 ,O3120X1 ,O3120X2 ,O3120X3 ,O3120X4 ,O3120X5 ,O3120X9 ,O3121X0 ,O3121X1<br>,O3121X2 ,O3121X3 ,O3121X4 ,O3121X5 ,O3121X9 ,O3122X0 ,O3122X1 ,O3122X2 ,O3122X3 ,O3122X4 ,O3122X5<br>,O3122X9 ,O3123X0 ,O3123X1 ,O3123X2 ,O3123X3 ,O3123X4 ,O3123X5 ,O3123X9 ,O3130X0 ,O3130X1 ,O3130X2<br>,O3130X3 ,O3130X4 ,O3130X5 ,O3130X9 ,O3131X0 ,O3131X1 ,O3131X2 ,O3131X3 ,O3131X4 ,O3131X5 ,O3131X9<br>,O3132X0 ,O3132X1 ,O3132X2 ,O3132X3 ,O3132X4 ,O3132X5 ,O3132X9 ,O3133X0 ,O3133X1 ,O3133X2 ,O3133X3<br>,O3133X4 ,O3133X5 ,O3133X9 ,O318X10 ,O318X11 ,O318X12 ,O318X13 ,O318X14 ,O318X15 ,O318X19 ,O318X20

© 2022 Admon LK et al. *JAMA Network Open.*

Pregnancy-associated hospitalizations	ICD-10
Antenatal complications continued	,O318X21 ,O318X22 ,O318X23 ,O318X24 ,O318X25 ,O318X29 ,O318X30 ,O318X31 ,O318X32 ,O318X33 ,O318X34<br>,O318X35 ,O318X39 ,O318X90 ,O318X91 ,O318X92 ,O318X93 ,O318X94 ,O318X95 ,O318X99 ,O3400 ,O3401 ,O3402<br>,O3403 ,O3410 ,O3411 ,O3412 ,O3413 ,O3430 ,O3431 ,O3432 ,O3433 ,O34593 ,O34599 ,O3460 ,O3461 ,O3462<br>,O3463 ,O3470 ,O3471 ,O3472 ,O3473 ,O3480 ,O3481 ,O3482 ,O3483 ,O3490 ,O3491 ,O3492 ,O3493 ,O350XX0<br>,O350XX1 ,O350XX2 ,O350XX3 ,O350XX4 ,O350XX5 ,O350XX9 ,O351XX0 ,O351XX1 ,O351XX2 ,O351XX3 ,O351XX4<br>,O351XX5 ,O351XX9 ,O352XX0 ,O352XX1 ,O352XX2 ,O352XX3 ,O352XX4 ,O352XX5 ,O352XX9 ,O353XX0 ,O353XX1<br>,O353XX2 ,O353XX3 ,O353XX4 ,O353XX5 ,O353XX9 ,O354XX0 ,O354XX1 ,O354XX2 ,O354XX3 ,O354XX4 ,O354XX5<br>,O354XX9 ,O355XX0 ,O355XX1 ,O355XX2 ,O355XX3 ,O355XX4 ,O355XX5 ,O355XX9 ,O356XX0 ,O356XX1 ,O356XX2<br>,O356XX3 ,O356XX4 ,O356XX5 ,O356XX9 ,O357XX0 ,O357XX1 ,O357XX2 ,O357XX3 ,O357XX4 ,O357XX5 ,O357XX9<br>,O358XX0 ,O358XX1 ,O358XX2 ,O358XX3 ,O358XX4 ,O358XX5 ,O358XX9 ,O359XX0 ,O359XX1 ,O359XX2 ,O359XX3<br>,O359XX4 ,O359XX5 ,O359XX9 ,O360110 ,O360111 ,O360112 ,O360113 ,O360114 ,O360115 ,O360119 ,O360120<br>,O360121 ,O360122 ,O360123 ,O360124 ,O360125 ,O360129 ,O360130 ,O360131 ,O360132 ,O360133 ,O360134<br>,O360135 ,O360139 ,O360190 ,O360191 ,O360192 ,O360193 ,O360194 ,O360195 ,O360199 ,O360910 ,O360911<br>,O360912 ,O360913 ,O360914 ,O360915 ,O360919 ,O360920 ,O360921 ,O360922 ,O360923 ,O360924 ,O360925<br>,O360929 ,O360930 ,O360931 ,O360932 ,O360933 ,O360934 ,O360935 ,O360939 ,O360990 ,O360991 ,O360992<br>,O360993 ,O360994 ,O360995 ,O360999 ,O361110 ,O361111 ,O361112 ,O361113 ,O361114 ,O361115 ,O361119<br>,O361120 ,O361121 ,O361122 ,O361123 ,O361124 ,O361125 ,O361129 ,O361130 ,O361131 ,O361132 ,O361133<br>,O361134 ,O361135 ,O361139 ,O361190 ,O361191 ,O361192 ,O361193 ,O361194 ,O361195 ,O361199 ,O361910<br>,O361911 ,O361912 ,O361913 ,O361914 ,O361915 ,O361919 ,O361920 ,O361921 ,O361922 ,O361923 ,O361924<br>,O361925 ,O361929 ,O361930 ,O361931 ,O361932 ,O361933 ,O361934 ,O361935 ,O361939 ,O361990 ,O361991<br>,O361992 ,O361993 ,O361994 ,O361995 ,O361999 ,O3620X0 ,O3620X1 ,O3620X2 ,O3620X3 ,O3620X4 ,O3620X5<br>,O3620X9 ,O3621X0 ,O3621X1 ,O3621X2 ,O3621X3 ,O3621X4 ,O3621X5 ,O3621X9 ,O3622X0 ,O3622X1 ,O3622X2<br>,O3622X3 ,O3622X4 ,O3622X5 ,O3622X9 ,O3623X0 ,O3623X1 ,O3623X2 ,O3623X3 ,O3623X4 ,O3623X5 ,O3623X9<br>,O365110 ,O365111 ,O365112 ,O365113 ,O365114 ,O365115 ,O365119 ,O365120 ,O365121 ,O365122 ,O365123<br>,O365124 ,O365125 ,O365129 ,O365130 ,O365131 ,O365132 ,O365133 ,O365134 ,O365135 ,O365139 ,O365190<br>,O365191 ,O365192 ,O365193 ,O365194 ,O365195 ,O365199 ,O365910 ,O365911 ,O365912 ,O365913 ,O365914<br>,O365915 ,O365919 ,O365920 ,O365921 ,O365922 ,O365923 ,O365924 ,O365925 ,O365929 ,O365930 ,O365931<br>,O365932 ,O365933 ,O365934 ,O365935 ,O365939 ,O365990 ,O365991 ,O365992 ,O365993 ,O365994 ,O365995<br>,O365999 ,O3660X0 ,O3660X1 ,O3660X2 ,O3660X3 ,O3660X4 ,O3660X5 ,O3660X9 ,O3661X0 ,O3661X1 ,O3661X2<br>,O3661X3 ,O3661X4 ,O3661X5 ,O3661X9 ,O3662X0 ,O3662X1 ,O3662X2 ,O3662X3 ,O3662X4 ,O3662X5 ,O3662X9<br>,O3663X0 ,O3663X1 ,O3663X2 ,O3663X3 ,O3663X4 ,O3663X5 ,O3663X9 ,O3670X0 ,O3670X1 ,O3670X2 ,O3670X3<br>,O3670X4 ,O3670X5 ,O3670X9 ,O3671X0 ,O3671X1 ,O3671X2 ,O3671X3 ,O3671X4 ,O3671X5 ,O3671X9 ,O3672X0<br>,O3672X1 ,O3672X2 ,O3672X3 ,O3672X4 ,O3672X5 ,O3672X9 ,O3673X0 ,O3673X1 ,O3673X2 ,O3673X3 ,O3673X4<br>,O3673X5 ,O3673X9 ,O3680X0 ,O3680X1 ,O3680X2 ,O3680X3 ,O3680X4 ,O3680X5 ,O3680X9 ,O368120 ,O368121<br>,O368122 ,O368123 ,O368124 ,O368125 ,O368129 ,O368130 ,O368131 ,O368132 ,O368133 ,O368134 ,O368135<br>,O368139 ,O368190 ,O368191 ,O368192 ,O368193 ,O368194 ,O368195 ,O368199 ,O368210 ,O368211 ,O368212

© 2022 Admon LK et al. *JAMA Network Open.*

**Pregnancy-associated hospitalizations**	**ICD-10**
Antenatal complications continued	,O368213 ,O368214 ,O368215 ,O368219 ,O368220 ,O368221 ,O368222 ,O368223 ,O368224 ,O368225 ,O368229<br>,O368230 ,O368231 ,O368232 ,O368233 ,O368234 ,O368235 ,O368239 ,O368290 ,O368291 ,O368292 ,O368293<br>,O368294 ,O368295 ,O368299 ,O368910 ,O368911 ,O368912 ,O368913 ,O368914 ,O368915 ,O368919 ,O368920<br>,O368921 ,O368922 ,O368923 ,O368924 ,O368925 ,O368929 ,O368930 ,O368931 ,O368932 ,O368933 ,O368934<br>,O368935 ,O368939 ,O368990 ,O368991 ,O368992 ,O368993 ,O368994 ,O368995 ,O368999 ,O3690X0 ,O3690X1<br>,O3690X2 ,O3690X3 ,O3690X4 ,O3690X5 ,O3690X9 ,O3691X0 ,O3691X1 ,O3691X2 ,O3691X3 ,O3691X4 ,O3691X5<br>,O3691X9 ,O3692X0 ,O3692X1 ,O3692X2 ,O3692X3 ,O3692X4 ,O3692X5,O3692X9 ,O3693X0 ,O3693X1 ,O3693X2<br>,O3693X3 ,O3693X4 ,O3693X5 , O3693X9 ,O401XX0 ,O401XX1 ,O401XX2 ,O401XX3 ,O401XX4 ,O401XX5 ,O401XX9<br>,O402XX0 ,O402XX1 ,O402XX2 ,O402XX3 ,O402XX4 ,O402XX5 ,O402XX9 ,O403XX0 ,O403XX1 ,O403XX2 ,O403XX3<br>,O403XX4 ,O403XX5 ,O403XX9 ,O409XX0 ,O409XX1 ,O409XX2 ,O409XX3 ,O409XX4 ,O409XX5 ,O409XX9 ,O4100X0<br>,O4100X1 ,O4100X2 ,O4100X3 ,O4100X4 ,O4100X5 ,O4100X9 ,O4101X0 ,O4101X1 ,O4101X2 ,O4101X3 ,O4101X4<br>,O4101X5 ,O4101X9 ,O4102X0 ,O4102X1 ,O4102X2 ,O4102X3 ,O4102X4 ,O4102X5 ,O4102X9 ,O4103X0 ,O4103X1<br>,O4103X2 ,O4103X3 ,O4103X4 ,O4103X5 ,O4103X9 ,O411010 ,O411011 ,O411012 ,O411013 ,O411014 ,O411015<br>,O411019 ,O411020 ,O411021 ,O411022 ,O411023 ,O411024 ,O411025 ,O411029 ,O411030 ,O411031 ,O411032<br>,O411033 ,O411034 ,O411035 ,O411039 ,O411090 ,O411091 ,O411092 ,O411093 ,O411094 ,O411095 ,O411099<br>,O411210 ,O411211 ,O411212 ,O411213 ,O411214 ,O411215 ,O411219 ,O411220 ,O411221 ,O411222 ,O411223<br>,O411224 ,O411225 ,O411229 ,O411230 ,O411231 ,O411232 ,O411233 ,O411234 ,O411235 ,O411239 ,O411290<br>,O411291 ,O411292 ,O411293 ,O411294 ,O411295 ,O411299 ,O411410 ,O411411 ,O411412 ,O411413 ,O411414<br>,O411415 ,O411419 ,O411420 ,O411421 ,O411422 ,O411423 ,O411424 ,O411425 ,O411429 ,O411430 ,O411431<br>,O411432 ,O411433 ,O411434 ,O411435 ,O411439 ,O411490 ,O411491 ,O411492 ,O411493 ,O411494 ,O411495<br>,O411499 ,O418X10 ,O418X11 ,O418X12 ,O418X13 ,O418X14 ,O418X15 ,O418X19 ,O418X20 ,O418X21 ,O418X22<br>,O418X23 ,O418X24 ,O418X25 ,O418X29 ,O418X30 ,O418X31 ,O418X32 ,O418X33 ,O418X34 ,O418X35 ,O418X39<br>,O418X90 ,O418X91 ,O418X92 ,O418X93 ,O418X94 ,O418X95 ,O418X99 ,O4190X0 ,O4190X1 ,O4190X2 ,O4190X3<br>,O4190X4 ,O4190X5 ,O4190X9 ,O4191X0 ,O4191X1 ,O4191X2 ,O4191X3 ,O4191X4 ,O4191X5 ,O4191X9 ,O4192X0<br>,O4192X1 ,O4192X2 ,O4192X3 ,O4192X4 ,O4192X5 ,O4192X9 ,O4193X0 ,O4193X1 ,O4193X2 ,O4193X3 ,O4193X4<br>,O4193X5 ,O4193X9 ,O43011 ,O43012 ,O43013 ,O43019 ,O43021 ,O43022 ,O43023 ,O43029 ,O43101 ,O43102<br>,O43103 ,O43109 ,O43111 ,O43112 ,O43113 ,O43119 ,O43121 ,O43122 ,O43123 ,O43129 ,O43191 ,O43192 ,O43193<br>,O43199 ,O43211 ,O43212 ,O43213 ,O43219 ,O43221 ,O43222 ,O43223 ,O43229 ,O43231 ,O43232 ,O43233 ,O43239<br>,O43811 ,O43812 ,O43813 ,O43819 ,O43891 ,O43892 ,O43893 ,O43899 ,O4390 ,O4391 ,O4392 ,O4393 ,O4400<br>,O4401 ,O4402 ,O4403 ,O4410 ,O4411 ,O4412 ,O4413 ,O4420 ,O4421 ,O4422 ,O4423 ,O4430 ,O4431 ,O4432 ,O4433<br>,O4440 ,O4441 ,O4442 ,O4443 ,O4450 ,O4451 ,O4452 ,O4453 ,O45001 ,O45002 ,O45003 ,O45009 ,O45011 ,O45012<br>,O45013 ,O45019 ,O45021 ,O45022 ,O45023 ,O45029 ,O45091 ,O45092 ,O45093 ,O45099 ,O458X1 ,O458X2 ,O458X3<br>,O458X9 ,O4590 ,O4591 ,O4592 ,O4593 ,O46001 ,O46002 ,O46003 ,O46009 ,O46011 ,O46012 ,O46013 ,O46019<br>,O46021 ,O46022 ,O46023 ,O46029 ,O46091 ,O46092 ,O46093 ,O46099 ,O468X1 ,O468X2 ,O468X3 ,O468X9 ,O4690<br>,O4691 ,O4692 ,O4693 ,O4700 ,O4702 ,O4703 ,O471 ,O479 ,O480 ,O481 ,O6000 ,O6002 ,O6003 ,O88011 ,O88012<br>,O88013 ,O88019 ,O88111 ,O88112 ,O88113 ,O88119 ,O88211 ,O88212 ,O88213 ,O88219 ,O88311 ,O88312 ,O88313<br>,O88319 ,O88811 ,O88812 ,O88813 ,O88819 ,O900 ,O901 ,O902 ,O905 ,O906 ,O91011 ,O91012 ,O91013 ,O91019

© 2022 Admon LK et al. *JAMA Network Open.*

Pregnancy-associated hospitalizations	ICD-10
Antenatal complications continued	,O91111 ,O91112 ,O91113 ,O91119 ,O91211 ,O91212 ,O91213 ,O91219 ,O98011 ,O98012 ,O98013 ,O98019 ,O98111 ,O98112 ,O98113 ,O98119 ,O98211 ,O98212 ,O98213 ,O98219 ,O98311 ,O98312 ,O98313 ,O98319 ,O98411 ,O98412 ,O98413 ,O98419 ,O98511 ,O98512 ,O98513 ,O98519 ,O98611 ,O98612 ,O98613 ,O98619 ,O98711 ,O98712 ,O98713 ,O98719 ,O98811 ,O98812 ,O98813 ,O98819 ,O98911 ,O98912 ,O98913 ,O98919 ,O99011 ,O99012 ,O99013 ,O99019 ,O99111 ,O99112 ,O99113 ,O99119 ,O99210 ,O99211 ,O99212 ,O99213 ,O99280 ,O99281 ,O99282 ,O99283 ,O99310 ,O99311 ,O99312 ,O99313 ,O99320 ,O99321 ,O99322 ,O99323 ,O99330 ,O99331 ,O99332 ,O99333 ,O99340 ,O99341 ,O99342 ,O99343 ,O99350 ,O99351 ,O99352 ,O99353 ,O99411 ,O99412 ,O99413 ,O99419 ,O99511 ,O99512 ,O99513 ,O99519 ,O99611 ,O99612 ,O99613 ,O99619 ,O99711 ,O99712 ,O99713 ,O99719 ,O99810 ,O99820 ,O99830 ,O99840 ,O99841 ,O99842 ,O99843 ,O9A111 ,O9A112 ,O9A113 ,O9A119 ,O9A211 ,O9A212 ,O9A213 ,O9A219 ,O9A311 ,O9A312 ,O9A313 ,O9A319 ,O9A411 ,O9A412 ,O9A413 ,O9A419 ,O9A511 ,O9A512 ,O9A513 ,O9A519
Maternal care	O320XX0 ,O320XX1 ,O320XX2 ,O320XX3 ,O320XX4 ,O320XX5 ,O320XX9 ,O321XX0 ,O321XX1 ,O321XX2 ,O321XX3 ,O321XX4 ,O321XX5 ,O321XX9 ,O322XX0 ,O322XX1 ,O322XX2 ,O322XX3 ,O322XX4 ,O322XX5 ,O322XX9 ,O323XX0 ,O323XX1 ,O323XX2 ,O323XX3 ,O323XX4 ,O323XX5 ,O323XX9 ,O324XX0 ,O324XX1 ,O324XX2 ,O324XX3 ,O324XX4 ,O324XX5 ,O324XX9 ,O326XX0 ,O326XX1 ,O326XX2 ,O326XX3 ,O326XX4 ,O326XX5 ,O326XX9 ,O328XX0 ,O328XX1 ,O328XX2 ,O328XX3 ,O328XX4 ,O328XX5 ,O328XX9 ,O329XX0 ,O329XX1 ,O329XX2 ,O329XX3 ,O329XX4 ,O329XX5 ,O329XX9 ,O330 ,O331 ,O332 ,O333XX0 ,O333XX1 ,O333XX2 ,O333XX3 ,O333XX4 ,O333XX5 ,O333XX9 ,O334XX0 ,O334XX1 ,O334XX2 ,O334XX3 ,O334XX4 ,O334XX5 ,O334XX9 ,O335XX0 ,O335XX1 ,O335XX2 ,O335XX3 ,O335XX4 ,O335XX5 ,O335XX9 ,O336XX0 ,O336XX1 ,O336XX2 ,O336XX3 ,O336XX4 ,O336XX5 ,O336XX9 ,O337XX0 ,O337XX1 ,O337XX2 ,O337XX3 ,O337XX4 ,O337XX5 ,O337XX9 ,O338 ,O339
Gestational age	Z3A00, Z3A01,Z3A08 ,Z3A09 ,Z3A10 ,Z3A11 ,Z3A12 ,Z3A13 ,Z3A14 ,Z3A15 ,Z3A16 ,Z3A17 ,Z3A18 ,Z3A19 ,Z3A20 ,Z3A21 ,Z3A22 ,Z3A23 ,Z3A24 ,Z3A25 ,Z3A26 ,Z3A27 ,Z3A28 ,Z3A29 ,Z3A30 ,Z3A31 ,Z3A32 ,Z3A33 ,Z3A34 ,Z3A35 ,Z3A36 ,Z3A37 ,Z3A38 ,Z3A39 ,Z3A40 ,Z3A41 ,Z3A42 ,Z3A49
Procedure codes	10D00Z0, 10D00Z1, 10D00Z2, 10D07Z3, 10D07Z4, 10D07Z5, 10D07Z6, 10D07Z7, 10D07Z8, 10E0XZZ
DRG	765, 766, 767, 768, 774, 775, 776, 769, 777, 778, 779, 770, 780, 781, 782 (for October 1, 2015- September 2018)<br>768, 783, 784, 785, 786, 787, 788, 796, 797, 798, 805, 806, 807, 769, 776, 777, 778, 779, 770, 780, 781, 782 (for October 1, 2018 – 2019)
**Delivery inclusion**	
Diagnosis codes	Z370, Z371, Z372, Z373, Z374, Z3750, Z3751, Z3752, Z3753, Z3754, Z3759, Z3760, Z3761, Z3762, Z3763, Z3764, Z3769, Z377, Z379, O80, O82, O7582
Procedure codes	10D00Z0, 10D00Z1, 10D00Z2, 10D07Z3, 10D07Z4, 10D07Z5, 10D07Z6, 10D07Z7, 10D07Z8, 10E0XZZ
DRG	765, 766, 767, 768, 774, 775 (for October 1, 2015- September 2018)<br>768, 783, 784, 785, 786, 787, 788, 796, 797, 798, 805, 806, 807 (for October 1, 2018 – 2019)
**Delivery exclusion**	
Diagnosis codes	O00, O01, O02, O03, O04, O07, O08
Procedure codes	10A00ZZ, 10A03ZZ, 10A04ZZ, 10A07Z6, 10A07ZW, 10A07ZX, 10A07ZZ, 10A08ZZ
**Postpartum**	
Diagnosis codes	

© 2022 Admon LK et al. *JAMA Network Open.*

**Pregnancy-associated hospitalizations**	**ICD-10**
Postpartum encounter	Z390,Z391,Z392
Maternal complications in the puerperium	O1003 ,O1013 ,O1023 ,O1033 ,O1043 ,O1093 ,O115 ,O1205 ,O1215 ,O1225 ,O135 ,O1405 ,O1415 ,O1425 ,O1495 ,O152 ,O165 ,O2403 ,O2413 ,O2433 ,O24430 ,O24434 ,O24435 ,O24439 ,O2483 ,O2493 ,O253 ,O2663 ,O2673 ,O870 ,O871 ,O872 ,O873 ,O874 ,O878 ,O879 ,O8803 ,O8813 ,O8823 ,O8833 ,O8883 ,O8901 ,O8909 ,O891 ,O892 ,O893 ,O894 ,O895 ,O896 ,O898 ,O899 ,O9081 ,O9089 ,O909 ,O9102 ,O9112 ,O9122 ,O9803 ,O9813 ,O9823 ,O9833 ,O9843 ,O9853 ,O9863 ,O9873 ,O9883 ,O9893 ,O9903 ,O9913 ,O99215 ,O99285 ,O99315 ,O99325 ,O99335 ,O99345 ,O99355 ,O9943 ,O9953 ,O9963 ,O9973 ,O99815 ,O99825 ,O99835 ,O99845 ,O9989 ,O9A13 ,O9A23 ,O9A33 ,O9A43 ,O9A53,O1033 ,O1043 ,O1093 ,O115 , O903, O904, O85, O860, O8611, O8612, O8613, O8619, O8620, O8621, O8622, O8629, O864, O8681,O8689
DRG	769, 776
**Antenatal**	All remaining pregnancy-associated hospitalizations not otherwise classified as delivery or postpartum

© 2022 Admon LK et al. *JAMA Network Open.*
